# Thermostable Basic Fibroblast Growth Factor Enhances the Production and Activity of Human Wharton’s Jelly Mesenchymal Stem Cell-Derived Extracellular Vesicles

**DOI:** 10.3390/ijms242216460

**Published:** 2023-11-17

**Authors:** SangRok Park, SeJong Kim, KyungMin Lim, YeoKyung Shin, Kwonwoo Song, Geun-Ho Kang, Dae Young Kim, Hang-Cheol Shin, Ssang-Goo Cho

**Affiliations:** 1 Department of Stem Cell and Regenerative Biotechnology, Molecular & Cellular Reprogramming Center and Institute of Advanced Regenerative Science, Konkuk University, 120 Neungdong-ro, Gwangjin-gu, Seoul 05029, Republic of Korea; packsangrok@naver.com (S.P.); rlatpwhdc@nate.com (S.K.); lmin0217@naver.com (K.L.); cutiesally@naver.com (Y.S.); rnjsdnthd814@naver.com (K.S.); geunhokang@naver.com (G.-H.K.); 2 R&D Team, StemExOne Co., Ltd., 307 KU Technology Innovation Bldg, 120 Neungdong-ro, Gwangjin-gu, Seoul 05029, Republic of Korea; 3 PnP Biopharm Co., Ltd., 1304, Acetechnotower 8-cha, 11 Digital-ro 33-gil, Guro-gu, Seoul 08380, Republic of Korea; kdypsh99@gmail.com (D.Y.K.); hcshin@pnpbiopharm.com (H.-C.S.)

**Keywords:** basic fibroblast growth factor, thermostable, Wharton’s jelly mesenchymal stem cell, exosome, wound healing, anti-inflammation

## Abstract

Wharton’s jelly-derived mesenchymal stem cell (WJ-MSC)-derived exosomes contain a diverse cargo and exhibit remarkable biological activity, rendering them suitable for regenerative and immune-modulating functions. However, the quantity of secretion is insufficient. A large body of prior work has investigated the use of various growth factors to enhance MSC-derived exosome production. In this study, we evaluated the utilization of thermostable basic fibroblast growth factor (TS-bFGF) with MSC culture and exosome production. MSCs cultured with TS-bFGF displayed superior proliferation, as evidenced by cell cycle analysis, compared with wild-type bFGF (WT-bFGF). Stemness was assessed through mRNA expression level and colony-forming unit (CFU) assays. Furthermore, nanoparticle tracking analysis (NTA) measurements revealed that MSCs cultured with TS-bFGF produced a greater quantity of exosomes, particularly under three-dimensional culture conditions. These produced exosomes demonstrated substantial anti-inflammatory and wound-healing effects, as confirmed by nitric oxide (NO) assays and scratch assays. Taken together, we demonstrate that utilization of TS-bFGF for WJ-MSC-derived exosome production not only increases exosome yield but also enhances the potential for various applications in inflammation regulation and wound healing.

## 1. Introduction

Mesenchymal stem cells (MSCs) are multipotent stem cells capable of self-renewal and differentiation into various cell types, including adipocytes, chondrocytes, osteoblasts, and cardiomyocytes [1,2]. MSCs secrete various inflammatory modulators, cytokines, and proteins involved in wound healing and inflammation control at the injury site and exhibit therapeutic effects regarding wound healing and anti-inflammatory responses. Therefore, MSCs have been the subject of research targeting numerous inflammatory conditions such as interstitial cystitis or bladder pain syndrome, arthritis and gastritis, and regeneration-related disorders, including nerve and muscle regeneration [3,4,5].

Wharton’s jelly-derived mesenchymal stem cells (WJ-MSCs) have garnered attention in regenerative medicine because of their unique advantages. These cells can be easily sourced from umbilical cord tissue, which is considered medical waste, thus bypassing ethical concerns [6]. Additionally, their superior proliferation and differentiation capabilities make them a promising alternative for various therapeutic applications (bone regeneration, neural regeneration, muscular regeneration, etc.) [7]. WJ-MSCs from umbilical cord tissue are less sensitive to oxidative changes, exhibit slower senescence, and have a higher proliferation rate as compared to MSCs derived from other tissue [8,9]. Furthermore, the immunomodulatory characteristics of WJ-MSCs make them attractive for cell-based therapies because of their low immunogenicity [10]. Previous studies have also highlighted the central role of WJ-MSCs in wound healing [11]. Despite the advantages of WJ-MSCs, limitations in clinical practice exist due to their potential for immunogenicity, renal and pulmonary consequences, and cancer risk [12]. 

Exosomes are small extracellular vesicles released by various cell types, including MSCs, and are involved in cell–cell communication in MSCs. Exosomes are crucial mediators of cell–cell communication because these vesicles contain various bioactive molecules, such as proteins, nucleic acids, and lipids, which can be transferred to recipient cells to modulate their function [5,13,14]. Cell culture conditions can influence the exosome secretion levels and functionality, thus enhancing their production yield and quality by optimizing culture conditions such as growth media composition [15,16,17,18]. Therefore, given the unique features of exosomes and their applicability, biotechnological fields have rapidly expanded by using exosomes as therapeutics, diagnostics, and cosmeceuticals [19,20,21,22,23]. However, despite their versatile applications in various fields, obtaining a sufficient quantity of exosomes remains a limitation. Various research efforts are underway to achieve a sufficient yield of EVs [5,10,13]. 

WJ-MSC-derived exosomes have garnered significant interest in regenerative medicine because of their therapeutic potential. In particular, MSC-derived exosomes are a treatment approach based on the paracrine mechanism of MSC secretion [24]. These extracellular vesicles (EVs) are bioactive molecule carriers and regulate various physiological processes [25,26]. WJ-MSCs promote cell proliferation and tissue regeneration, key elements in tissue repair and wound healing [27]. Furthermore, exosomes exhibit remarkable anti-inflammatory properties [28]. Exosomes contain numerous immunomodulatory factors that can mitigate inflammatory responses, making them promising tools for managing inflammatory disorders [29]. Therefore, the immunomodulatory abilities and wound-healing properties of WJ-MSC exosomes highlight their potential for different treatment modalities [30].

Based on our previous study with thermostable basic fibroblast growth factor (TS-bFGF) on pluripotent stem cells (PSCs) [31], in this study, we aimed to further expand our understanding of the effect of TS-bFGF on MSCs. This study demonstrates that MSCs cultured with TS-bFGF had better characteristics regarding cell proliferation, stemness, and exosome secretion levels and enhanced effects on wound healing and anti-inflammatory effects. Therefore, TS-bFGF can be applied to broader stem cell types in various cell culture processes, producing high-quality stem cells and exosomes.

## 2. Results

### 2.1. Characterization of WJ-MSCs Treated with Wild-Type bFGF or TS-bFGF 

**Figure 1:**
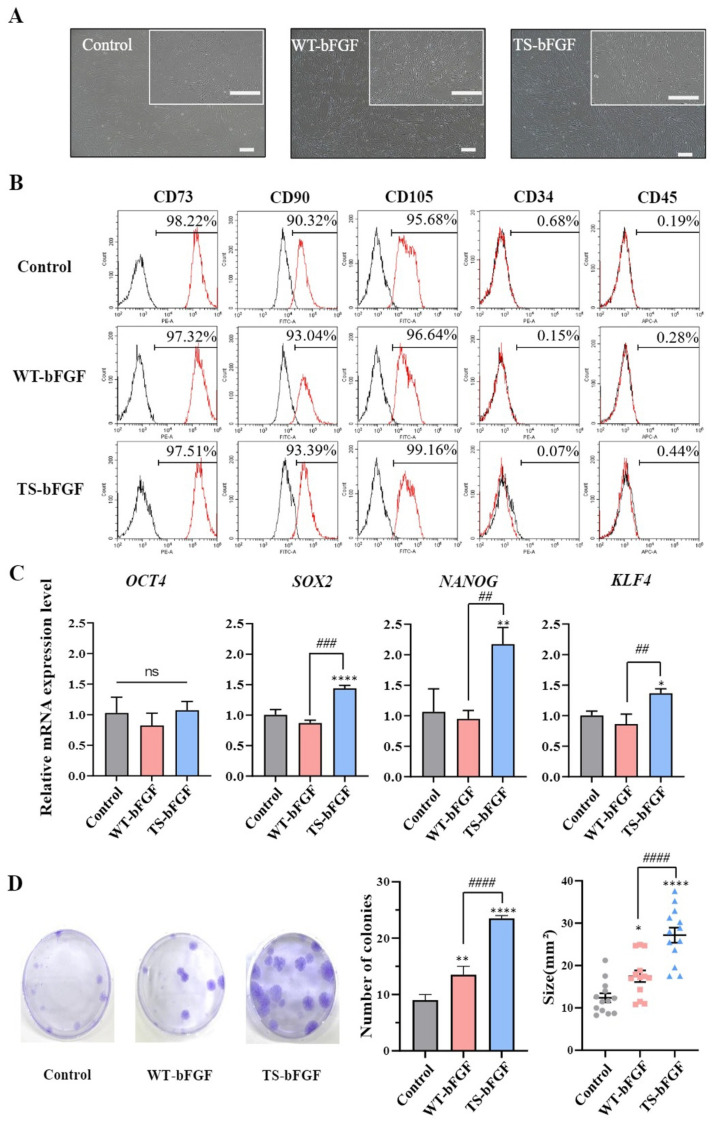
Characterization of Wharton’s jelly-derived mesenchymal stem cells (WJ-MSCs) after treatment with wild-type basic fibroblast growth factor (WT-bFGF) or thermostable bFGF (TS-bFGF). (**A**) Phase-contrast microscopy images of WJ-MSCs and WJ-MSCs treated with WT-bFGF (WT-WJ-MSCs) and WJ-MSCs treated with TS-bFGF (TS-WJ-MSCs). Scale bar: 200 μm. (**B**) Flow cytometry analysis of positive marker (CD73, CD90, and CD105) and negative marker (CD34 and CD45) expression in WT-WJ-MSCs and TS-WJ-MSCs. (**C**) *OCT4*, *SOX2*, *NANOG*, and *KLF4* gene expression levels in WT-WJ-MSCs and TS-WJ-MSCs over 3 days. The mRNA expression levels were determined using quantitative RT-PCR and normalized to the control group. (**D**) Fibroblast colony-forming unit (CFU-F) assays were performed to assess CFU-F formation in WT-WJ-MSCs and TS-WJ-MSCs, and the results are presented in photographs and graphs. The data are presented as the mean ± standard deviation (n = 3). ns: not significant. * *p* < 0.05, ** *p* < 0.01, and **** *p <* 0.0001 compared to the control group and ## *p* < 0.01, ### *p* < 0.001, and #### *p* < 0.0001 in comparison to the WT-3D EV group.

This study aimed to assess the impact of TS-bFGF treatment on the characteristics of WJ-MSCs compared with wild-type bFGF (WT-bFGF). Initially, we confirmed that the morphological features of WJ-MSCs remained unaltered following treatment with either WT-bFGF or TS-bFGF (Figure 1A). Furthermore, flow cytometry analysis revealed that WJ-MSCs treated with either WT-bFGF or TS-bFGF continued to express stem cell markers, notably CD73 and CD90, while maintaining negative expression of differentiation markers such as CD34 and CD45 (Figure 1B). These findings suggest that treatment with WT-bFGF or TS-bFGF does not seem to impact the preservation of stemness characteristics in WJ-MSCs.

To further compare the stemness attributes of WJ-MSCs treated with the two bFGF variants, we conducted quantitative RT-PCR (qRT-PCR) to assess the expression of key genes essential for maintaining stemness, including *OCT4*, *SOX2*, *NANOG*, and *KLF4*. *OCT4* expression levels remained similar in WJ-MSCs treated with either WT-bFGF or TS-bFGF. However, WJ-MSCs treated with TS-bFGF (TS-WJ-MSCs) exhibited increased expression of *SOX2*, *NANOG*, and *KLF4* compared to those treated with WT-bFGF (WT-WJ-MSCs), suggesting that TS-WJ-MSCs likely retained their stemness characteristics more effectively (Figure 1C). 

We also verified the colony-forming ability of both WT-WJ-MSCs and TS-WJ-MSCs by quantifying fibroblast colony-forming unit (CFU-F) values. TS-WJ-MSCs demonstrated a significantly higher CFU-F value compared to that of WT-WJ-MSCs (Figure 1D), indicating that TS-bFGF was superior to WT-bFGF in terms of maintaining stemness characteristics in WJ-MSCs and promoting their growth. These results demonstrated that TS-WJ-MSCs exhibit superior stemness maintenance compared to WT-WJ-MSCs.

### 2.2. TS-bFGF Increases Cell Proliferation

**Figure 2:**
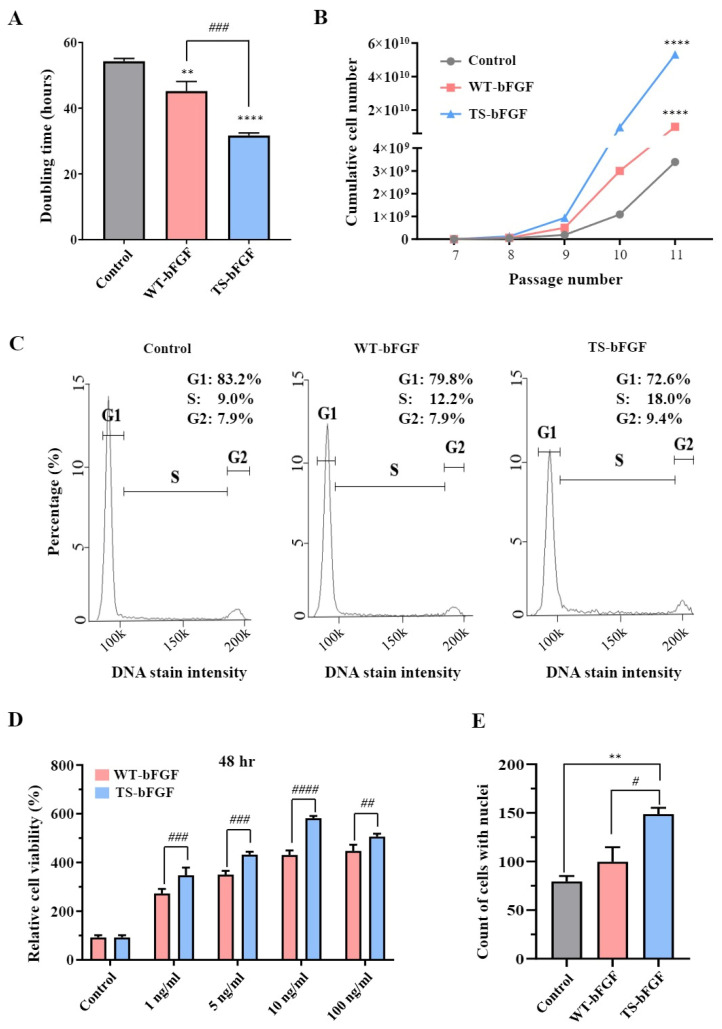
Cell proliferation in WT-WJ-MSCs and TS-WJ-MSCs. (**A**,**B**) Comparison of doubling time (**A**) and cumulative cell numbers (**B**) in continuous cell culture with media change every 2–3 days for WT-WJ-MSCs and TS-WJ-MSCs. (**C**) Histogram analysis of cell cycle distribution in WT-WJ-MSCs and TS-WJ-MSCs. The x-axis represents the cell cycle phases (G1, S, and G2), and the y-axis represents the percentage of cells in each phase. (**D**) After 48 h of treatment, cell viability was measured with different WT-bFGF or TS-bFGF concentrations (1, 5, 10, and 100 ng/mL). The cell viability values were normalized by setting the control values as 100%. (**E**) Nuclei were counted using 4′6-diamidino-2-phenylindole (DAPI) staining after 2 days of incubation with media containing WT-bFGF or TS-bFGF. The values are presented as the mean ± standard error of the mean (SEM) from three independent experiments. Statistical significance was determined using a *t*-test. ** *p* < 0.01 and **** *p <* 0.0001 compared to the control group and # *p* < 0.05, ## *p* < 0.01, ### *p* < 0.001, and #### *p* < 0.0001 is comparison with the WT-bFGF group.

We investigated the effects of WT-bFGF and TS-bFGF on the growth of WJ-MSCs treated with TS-bFGF compared to WT-bFGF regarding cell proliferation. Notably, TS-WJ-MSCs showed a significantly shorter reduced doubling time compared to the WT-WJ-MSCs (Figure 2A), indicating that TS-bFGF accelerates cell division and proliferation, leading to a more rapid increase in cell numbers. Furthermore, TS-WJ-MSCs exhibited a substantial increase in cumulative cell numbers when compared to WT-WJ-MSCs (Figure 2B). 

Moreover, cell cycle analysis showed that TS-WJ-MSCs had more cells in the S phase, suggesting that TS-bFGF promotes cell cycle progression and increases cell growth. We also investigated the effects of TS-bFGF on cell proliferation by examining various bFGF concentrations. Cell proliferation was significantly higher in TS-WJ-MSCs than in WT-WJ-MSCs (Figure 2D and Figure S1) at different WT-bFGF and TS-bFGF concentrations (1, 5, 10, and 100 ng/mL). This result was further supported by visualizing nuclear division using 4′6-diamidino-2-phenylindole (DAPI) staining, where TS-WJ-MSCs exhibited a higher number of nuclei than WT-WJ-MSCs (Figure 2E and Figure S2). These results demonstrated that TS-bFGF outperformed WT-bFGF in WJ-MSC proliferation.

### 2.3. Characterization of WT-WJ-MSCs- and TS-WJ-MSCs-Derived Exosomes

**Figure 3:**
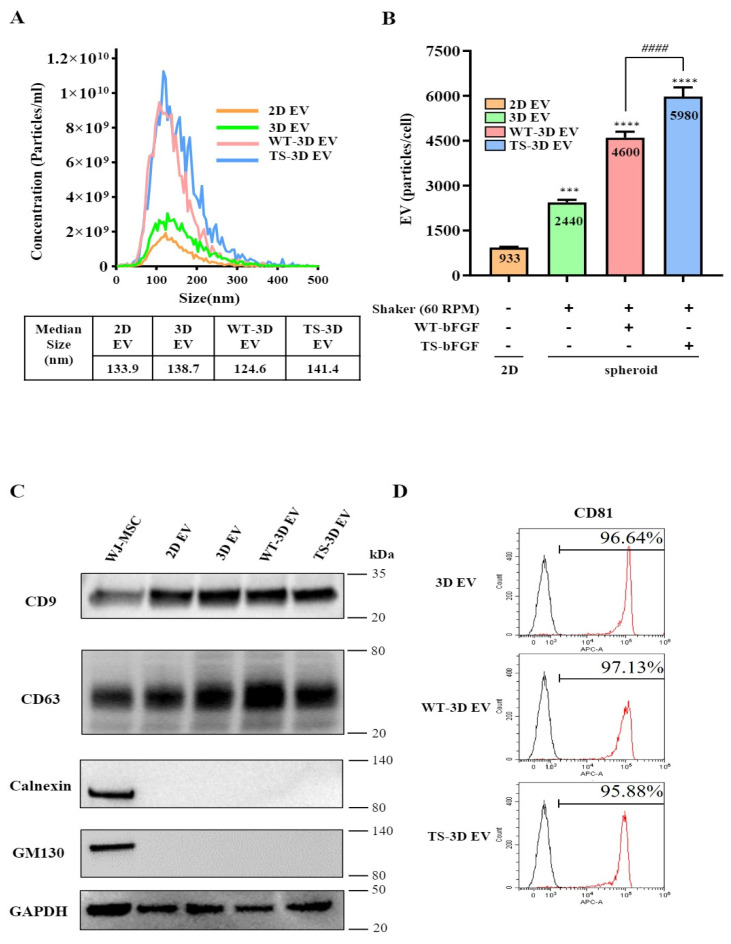
Characterization of WJ-MSC-derived exosomes treated with WT-bFGF (WT-3D EVs) or TS-bFGF (TS-3D EVs). (**A**) The particle size comparison was carried out using ZetaView. The concentration is indicated as particles/mL. Two-dimensional EV and 3D EV: EVs derived from WJ-MSCs cultured under 2D or 3D conditions, respectively. (**B**) Comparison of exosome production. Increased exosome production was observed with TS-bFGF treatment compared to WT-bFGF treatment under the same culture conditions. (**C**) Expression analysis of CD63 and CD9 (positive exosome markers) and GM130 and calnexin (negative exosome markers) using Western blotting. CD63 and CD9 expressions were detected in all exosome groups. (**D**) Analysis of CD81 expression in exosomes isolated using exosome human CD9 beads. *** *p* < 0.01, **** *p* < 0.001 compared to the control group. #### *p* < 0.0001 compared to the WT-3D EV group.

To investigate the impact of TS-bFGF on WJ-MSC-derived exosomes, we explored their production under three-dimensional (3D) culture conditions, comparing them to two-dimensional (2D) cultured WJ-MSCs. We employed a nanoparticle tracking analyzer (NTA) to assess the size of EVs generated by WJ-MSCs treated with either WT-bFGF (WT-3D EVs) or TS-bFGF (TS-3D EVs) in 3D culture. The analysis revealed that the size of the particles fell within the range of 50 to 150 nm, indicating that bFGF treatment did not significantly alter EV size (Figure 3A). Furthermore, TS-bFGF treatment led to a substantial increase in EV production compared to WT-bFGF treatment, in which there were substantially higher numbers of EVs in conditioned media derived from TS-3D WJ-MSCs compared to WT-3D WJ-MSCs (Figure 3B and Supplementary Table S1). To assess the impact of bFGF treatment on EV characteristics, we conducted a Western blot analysis. The results indicated that the essential features of EVs remained unaltered following bFGF treatment. We detected EV-positive markers, including tetraspanin proteins (CD9 and CD63), in both WT-3D EVs and TS-3D EVs, while EV-negative markers such as Golgi protein (GM130) and endoplasmic reticulum protein (calnexin) were absent in the isolated EVs (Figure 3C). Flow cytometry analysis further confirmed the presence of CD81, another exosome marker, in WT-3D EVs and TS-3D EVs (Figure 3D). In summary, our findings demonstrate that TS-bFGF enhances EV production in WJ-MSCs within a 3D culture environment when compared to WT-bFGF, while maintaining EV characteristics, including size and exosomal marker expression.

### 2.4. TS-3D EVs Increased Cell Proliferation and Wound Closure Compared to WT-3D EVs

**Figure 4:**
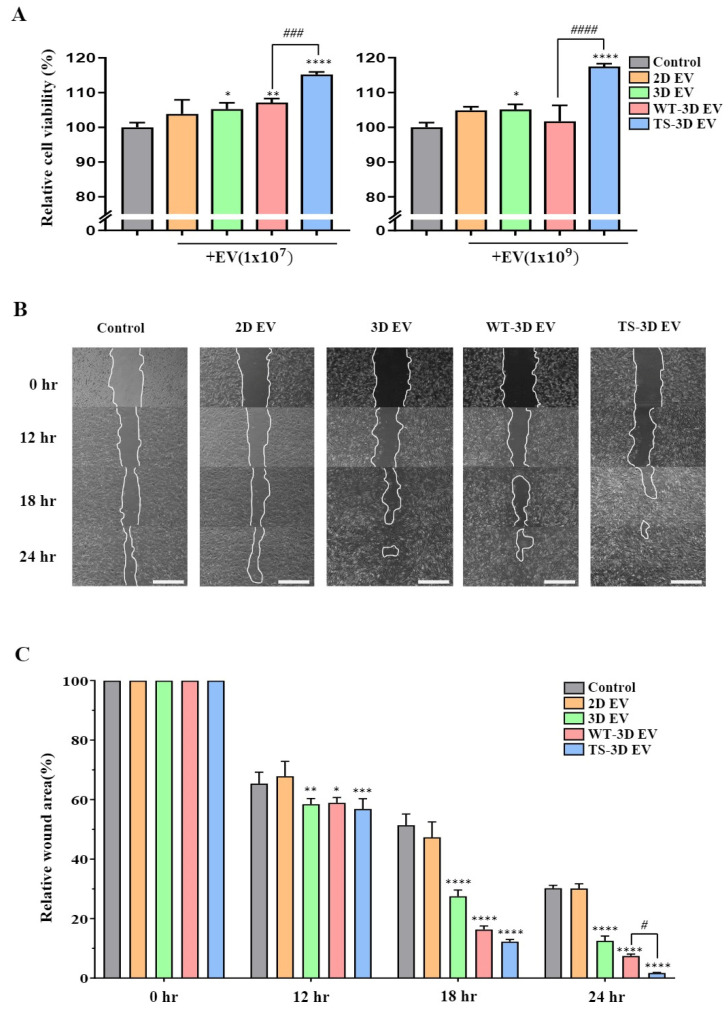
Characterization of EVs in cell proliferation and wound healing. (**A**) The cell proliferation rate of human dermal fibroblasts (HDFs) treated with each EV at 1 × 10^7^ and 1 × 10^9^ particles/mL. (**B**) Images showing the wound-healing process in HDFs treated with different EVs after the scratch test and cultured various times. Scale bar: 200 μm. (**C**) Bar graph showing the relative wound area (setting 0 h as 100%) by time points in each group shown in (**B**). Statistical significance is denoted as * *p* < 0.05, ** *p* < 0.01, *** *p* < 0.001, and **** *p <* 0.0001 compared to the control group, and # *p* < 0.05, ### *p* < 0.001, and #### *p* < 0.0001 compared to the WT-3D EV groups.

We conducted an investigation into the impact of WT-3D EVs and TS-3D EVs on cell viability and wound healing. The cell viability assay indicated that TS-3D EVs notably enhanced cell viability in comparison to the other EV types (2D, 3D, and WT-3D EVs) at both low and high concentrations (1 × 10^7^ particles/mL and 1 × 10^9^ particles/mL, respectively) (Figure 4A). Furthermore, we assessed the wound-healing effects using a wound-closure assay. TS-3D EVs demonstrated significantly accelerated closure of the wound site in comparison to other EVs (Figure 4B,C). These findings strongly suggest that TS-3D EVs exhibit higher efficiency in promoting wound closure than WT-3D EVs, likely attributable to their heightened cell proliferation activity.

### 2.5. Anti-Inflammatory Activity of EVs from WT-WJ-MSCs and TS-WJ-MSCs

**Figure 5:**
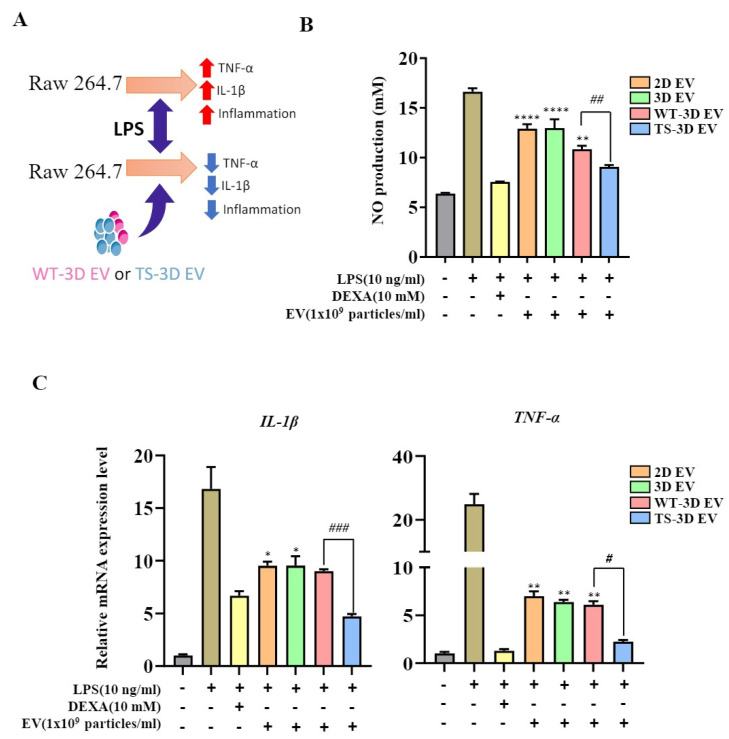
Characterization of WT-3D EVs and TS-3D EVs in anti-inflammation activity. (**A**) Graphical abstract illustrating the anti-inflammatory effects of WT-3D EVs and TS-3D EVs in RAW 264.7 cells. (**B**) Measurement of nitric oxide (NO) levels in RAW 264.7 cells co-cultured with lipopolysaccharide (LPS; 10 ng/mL) and EVs (1 × 10^9^) for 24 h using the Griess assay. (**C**) Comparison of gene expression levels of pro-inflammatory cytokines in RAW 264.7 cells treated with EVs in the presence of LPS. The expression levels of pro-inflammatory cytokines were analyzed using qRT-PCR. Statistical significance was determined using * *p* < 0.05, ** *p* < 0.01, and **** *p* <0.0001 compared to the LPS and DEXA-treated control group. # *p* < 0.05, ## *p* < 0.01 and ### *p* < 0.001 compared to the WT-3D EV group. All experiments were performed independently in triplicate. IL, interleukin; TNF, tumor necrosis factor.

We investigated the anti-inflammatory activity of EVs, specifically comparing WT-3D EVs and TS-3D EVs. We used a nitric oxide (NO) assay to measure the amount of NO expressed in LPS-treated RAW264.7 cells in response to molecules such as EVs. TS-3D EVs exhibited a significantly greater reduction in NO levels compared to other EVs (Figure 5B). Additionally, RNA expression levels of the inflammatory cytokines TNF-α and IL-1β were assessed. Both WT-3D EVs and TS-3D EVs reduced the expression of these cytokines, with TS-3D EVs showing a more pronounced decrease as compared to WT-3D EVs.

## 3. Discussion

Growth factors such as bFGF are crucial for tissue development and maintenance [32]. TS-bFGF is more stable than WT-bFGF and is more beneficial in enhancing overall cellular functions, such as maintaining pluripotency and promoting self-renewal capacity, primarily in PSCs [31]. Since research on TS-bFGF has been predominantly conducted on its culture outcomes with PSCs, such as embryonic stem cells (ESCs) and induced pluripotent stem cells (iPSCs), few studies have explored its functional enhancement in other cell types, such as MSCs. To address this limitation, we investigated the impact of TS-bFGF treatment on the characteristics and proliferative abilities of WJ-MSCs, which are well known for their regenerative and immunomodulatory properties and have been extensively researched as promising cellular therapeutic strategies. 

Our study provides new insights into the role of TS-bFGF in WJ-MSCs by comparing them with WT-bFGF in terms of cell growth dynamics and exosomal characteristics. As observed previously in PSCs, TS-bFGF demonstrated superior cell culture outcomes compared to WT-bFGF in WJ-MSCs [31]. WJ-MSCs treated with WT-bFGF or TS-bFGF expressed MSC markers (CD73 and CD90), indicating that bFGF treatment did not induce unwanted differentiation or alterations in WJ-MSC identity, critical for potential therapeutic applications. In addition, key stemness genes, such as *SOX2*, *NANOG*, and *KLF4*, which are critical for stemness maintenance, were significantly upregulated in WJ-MSCs treated with TS-bFGF compared to WT-bFGF, suggesting that TS-bFGF enhances stemness maintenance in WJ-MSCs, consistent with our previous results on PSCs [31]. Maintaining the stemness of MSCs is crucial for preserving their characteristics [33]. As previously established, WT-bFGF plays a significant role not only in the growth and proliferation of stem cells but also in maintaining their stemness [34]. According to our previous research findings, the expression of TS-bFGF was observed to be sustained for a longer duration compared to WT-bFGF [31]. However, further investigations are needed to elucidate the pathway between TS-bFGF and stem cell genes (4 October, SOX2, etc.). Such research will contribute to enhancing the maintenance of stemness in MSCs. The reason for this is unclear, and further study is needed to elucidate the cause of the difference in gene expression between the two stem cells. As observed in iPSCs treated with TS-bFGF, TS-bFGF-treated WJ-MSCs also exhibited superior colony-forming ability compared to WT-bFGF, suggesting that TS-bFGF enhanced the self-renewal capability of WJ-MSCs. 

We also investigated whether TS-bFGF could exert a stimulatory effect on cell proliferation in WJ-MSCs based on previous studies indicating the positive role of bFGF in cell proliferation [35,36,37] and previous findings on ESCs. As expected, TS-bFGF showed better cell proliferation outcomes than WT-bFGF. TS-bFGF significantly reduced the cell doubling time (i.e., faster cell duplication), resulting in higher cumulative cell numbers over the cell culture period compared to WT-bFGF, consistent with our previous results in PSCs. This finding was supported by cell cycle analysis and cell counting coupled with DAPI staining; when WJ-MSCs were treated with TS-bFGF, more cells were in the S phase, and more DAPI-stained cells were present compared to WT-bFGF-treated conditions. These results indicate that TS-bFGF enhanced cell cycle progression and increased nuclear division, allowing WJ-MSCs to divide more rapidly, resulting in higher cumulative cell numbers [35,36]. However, further studies will reveal more details regarding the crosstalk between TS-bFGF and cell cycle regulators, enabling a better understanding of the role of TS-bFGF in cell proliferation.

Exosomes are small extracellular vesicles that play a crucial role in cell-to-cell communication by delivering proteins, lipids, and nucleic acid cargo from donor to recipient cells. WJ-MSCs possess various differentiation, self-renewal, and paracrine regulatory functions [38,39]. Paracrine signaling and therapeutic efficacy may be affected because enhanced exosome release has also been reported in WJ-MSC-derived exosomes carrying bioactive molecules that influence various biological processes, including immunomodulation, tissue regeneration, and angiogenesis [11,40]. This study elucidated the effects of bFGF treatment on the production and characteristics of EVs derived from WJ-MSCs. Our research findings support previous reports of increased exosome production in 3D-cultured MSCs compared to 2D culture and demonstrate the enhancing effect of TS-bFGF on exosome production [5,41].

Despite increased EV production, the EV characteristics were unchanged upon TS-bFGF treatment. The size distributions of TS-3D EVs and WT-3D EVs were not significantly altered. This result is important because the vesicle size can influence biodistribution, cellular uptake, and cargo capacity. In addition, no change in the characteristic protein markers, such as CD81, present on the surface of TS-3D EVs or WT-3D EVs, was observed [3,13], another important aspect in the quality control of EVs; otherwise, monitoring the quality of EVs would be challenging. The cause of increased EV production is unclear; however, the increased EV production might result from enhanced cell proliferation induced by TS-bFGF rather than WT-bFGF, which requires further study. Maximizing the production yield by optimizing the culture conditions (i.e., optimizing culture media and different culture methods, such as a 3D suspension culture) for industrial-scale production of EVs is essential. 

Exosomes can be used as a therapeutic agent owing to their ability to carry bioactive molecules, such as growth factors and miRNAs, which can modulate various biological processes [42,43,44]. Since WJ-MSCs treated with TS-bFGF had improved characteristics, we reasoned that TS-3D EVs could also have superior activity compared to WT-3D EVs. As expected, significant enhancement of cell viability was observed with TS-3D EVs compared to WT-3D EVs. This finding indicates that TS-bFGF can produce exosomes possessing a superior cargo profile to promote cell survival and proliferation more effectively than WT-bFGF. The wound closure assay revealed significantly more efficient wound healing with TS-3D EVs than with WT-3D EVs. Additionally, WT-bFGF regulates calcium homeostasis [45], and research has associated the generation of exosomes with the Ca^2+^ pathway [46,47]. Further investigation into the Ca^2+^ pathway is required to understand the relationship between TS-bFGF and exosome production. As WJ-MSCs treated with TS-bFGF have increased cell proliferation activity, they could inherit more regenerative molecules to TS-3D EVs. Comparing TS-3D EVs- and WT-3D EVs by characterizing their components using biochemical tools such as RNAseq and proteomic analysis would be interesting. 

The role of TS-3D EVs was explored based on previous studies demonstrating the anti-inflammatory effects of WJ-MSC-derived EVs [48,49,50]. A stronger anti-inflammatory effect was observed in TS-3D EVs compared to WT-3D EVs. In addition, a greater reduction in the RNA expression levels of pro-inflammatory cytokines, such as *TNF-α* and *IL-1β*, was observed with TS-3D EVs than with WT-3D EVs. This finding suggests that TS-bFGF may induce changes in the cargo content or functional properties of WJ-MSC-derived EVs, leading to an enhanced anti-inflammatory effect. However, biochemically characterizing EVs is crucial to better understand the anti-inflammatory effects of TS-3D EVs compared with those of WT-3D EVs. Thus, optimizing TS-3D EVs further to harness their potential benefits for cell-based therapies to promote tissue regeneration and anti-inflammatory applications is possible. While our study highlights the potential benefits of TS-bFGF in cell growth and exosome production, further research is needed to fully understand the underlying cellular pathways and fundamental mechanisms involved.

In conclusion, we demonstrated that TS-bFGF outperforms WT-bFGF in WJ-MSC cell growth dynamics, and TS-3D EVs show better outcomes in cell viability, wound healing, and anti-inflammation compared to WT-3D EVs. These results further expand our understanding of TS-bFGF-derived PSCs on WJ-MSCs in cell growth dynamics and exosomal characteristics. Therefore, TS-bFGF can be applied to more diverse stem cell types, leading to high-quality stem cells and EVs that could be developed as therapeutics and biological materials in tissue engineering, regenerative medicine, and anti-inflammatory treatments.

## 4. Materials and Methods

### 4.1. Cell Culture

The WJ-MSCs were isolated according to the method described in our previous study (IRB no. 7001355-202010-BR-407). The cells were grown in α-minimum essential medium (α-MEM, 12561072, Gibco, Waltham, MA, USA) with a 10% concentration of fetal bovine serum (FBS, PS-FB1, PEAK) and 1% penicillin/streptomycin (P/S; 15140-163, Gibco) in a humidified incubator at 37 °C with 5% CO_2_. RAW 264.7 cells and human dermal fibroblasts (HDFs) were cultivated in high-glucose Dulbecco’s modified Eagle medium (DMEM, D6429, Sigma-Aldrich, St. Louis, MO, USA) enriched with 10% FBS and 1% P/S.

### 4.2. Flow Cytometry

Three days after culturing the WJ-MSCs with 10 ng/μL of WT-BbFGF or TS-bFGF, the cells were detached with 0.25% trypsin-EDTA (25200-56, Gibco) and resuspended in 1× PBS containing 2% FBS. The primary antibody was then reacted at 4 °C for 90 min, followed by centrifugation at 1200 rpm for 5 min, and washed thrice with 1× PBS. The cell pellet of the primary antibody was then reacted with the secondary antibody at 4 °C for 1 h and 30 min under the same conditions. The fluorescence intensity of the antibodies was measured using flow cytometry (CytoFLEX, Beckman Coulter, Brea, CA, USA). The primary antibodies used were against CD34 (130–117-775, Miltenyi Biotec, Cologne, Germany), CD45 (130-110-771, Miltenyi Biotec, Cologne, Germany), CD73 (41-0200, Invitrogen, Waltham, MA, USA, A-10680), CD90 (AF2067, R&D Systems, Minneapolis, MN, USA), and CD105 (MA5–11854, Invitrogen). Goat anti-mouse IgG (A28175; Invitrogen) and donkey anti-sheep IgG (ab7009; Abcam) were used as secondary antibodies. EV surface markers were measured using fluorescence-activated cell sorting. After treatment with WT-bFGF and TS-bFGF, 1 × 10^9^ EV particles extracted from the WJ-MSCs were reacted with CD9 magnetic beads (10620D, Invitrogen) at 4 °C for one day. After washing thrice with filtered PBS, the secondary antibodies were CD63-PE (556020, BD Pharmingen) and CD81-APC (130–119-787, Miltenyi Biotec). Antibody-labeled EVs were measured using flow cytometry (CytoFLEX, Beckman Coulter).

### 4.3. Quantitative RT-PCR

Total RNA was isolated from the WJ-MSCs and RAW 264.7 cells using Labozol Reagent (CMRZ001, Labopass, Seoul, Republic of Korea) according to the manufacturer’s instructions. RNA was analyzed using a Nanodrop spectrophotometer (IMPLEN, Müchen, Germany). For cDNA synthesis, 2 μg of RNA was transduced into HiPi Real-Time PCR 2× Master Mix (SYBR green, ROX) (EBT-1802, ELPISBIO, Daejeon, Republic of Korea) and used for RT-PCR. Gene expression was normalized to the housekeeping GAPDH gene and calculated using the ΔΔCt method. The primers used in this study are listed in Table 1.

### 4.4. Fibroblast Colony-Forming Unit Assay

The WJ-MSCs were seeded at a density of 30 cells/cm^2^ in each 60 mm dish containing WT-bFGF or TS-bFGF, respectively. After 14 days, the cells were washed with 1× PBS and fixed/stained using a methanol-containing fixation/staining solution (0.5% crystal violet aqueous solution/methanol, ratio of 1:1) at room temperature for 15 minutes (V5265, Sigma, Seoul, Republic of Korea). Subsequently, the cells were rinsed three times with distilled water. An aggregate of more than 50 cells was defined as a CFU-F.

### 4.5. Cell Growth Kinetics

The proliferation of WJ-MSCs treated with WT-bFGF or TS-bFGF individually was determined by calculating the cumulative cell number (CN) based on continuous passaging and cell growth. The CN was calculated using the following equation CN = $\ln\left(N_{f}/N_{i}\right)$/ln⁡2, where $N_{i}$ represents the initial cell number and $N_{f}$ represents the final cell number. The natural logarithm (ln) was used in the calculation. WJ-MSCs were cultured in a 100 mm dish with an initial seeding of 2.88 × 10^5^ cells (n = 3). When confluency reached 70–80%, the cell count was measured before passaging for further cultivation. To determine the cumulative population doubling level, each segment’s population doubling level was calculated and added to the previous population doubling level. WT-bFGF or TS-bFGF were treated every 2 to 3 days.

### 4.6. Cell Cycle Assay

We used a NucleoCounter^®^ NC-250™ (ChemoMetec, Allerod, Denmark) following the manufacturer’s instructions to assess cell division and expansion. The cells were seeded at a density of 5 × 10^3^ cells/cm^2^ on a 60 mm dish and treated with WT-bFGF or TS-bFGF. The cells were harvested after 24 h, washed with 1× PBS, and resuspended in a DAPI solution (910-3012, ChemoMetec). A stabilizing buffer was added after incubation for 5 min at 37 °C. We measured cell division and expansion using the NucleoCounter^®^ NC-250™ (ChemoMetec) following the manufacturer’s instructions. The cells were seeded onto a 60 mm dish at a density of 5 × 10^3^ cells/cm^2^ and treated with WT-bFGF or TS-bFGF. After 24 h, the cells were collected, washed with 1× PBS, and resuspended in a DAPI solution. After 5-min 37 °C incubation, a stabilizing buffer was added, and the NucleoCounter (NC-250) was used to measure cell division and expansion.

### 4.7. Cell Proliferation Test

We plated 5 × 10^3^ cells in 96-well plates (30096, SPL, Seoul, Republic of Korea) to assess the effects of bFGF on the WJ-MSC culture. The cells were treated with WT-bFGF and TS-bFGF at 1, 5, 10, or 100 ng/mL concentrations. The culture medium comprised α-MEM supplemented with 10% FBS and 1% P/S. The medium was replaced with the reagents provided in the viability assay kit (B1007–500, Cellrix, Seoul, Republic of Korea) at 24 h and 48 h after cell seeding. The growth rates of the WT-bFGF- and TS-bFGF-treated cells were evaluated by measuring the absorbance at 450 nm using a Bio-RADx-MarkTM spectrophotometer. WT-bFGF and TS-bFGF were incubated at 4 °C and 37 °C for 72 h, respectively. The proteins were stored aseptically in tubes until further use. WJ-MSC and HDF were seeded in individual wells of a 96-well plate. The seeded cells were treated with the incubated WT-bFGF and TS-bFGF at a 10 μg/mL concentration at 4 °C and 37 °C. After 24 h of bFGF treatment, cell proliferation was assessed using spectrophotometry. The absorbance of each well was measured at 450 nm to determine the cell proliferation rate in the bFGF-treated group. The cell proliferation rate was measured after treating the HDFs with EVs produced by bFGF. Each well was seeded at 5 × 10^3^ cells, and EVs were added at a concentration of 1 × 10^7^ or 1 × 10^9^ particles/mL. The absorbance was measured at 450 nm using an x-MarkTM spectrophotometer (Bio-Rad Laboratories, Hercules, CA, USA) after incubation for 24 h.

### 4.8. DAPI Staining Assay

For fluorescent staining, 10,000 WJ-MSCs in a 60 mm dish were treated with WT-bFGF and TS-bFGF and cultured for 2 days. After removing the culture medium, the WJ-MSCs were washed thrice with 1× PBS and fixed with 4% paraformaldehyde. Subsequently, a DAPI solution diluted in methanol to a concentration of 1 μg/mL was prepared. Each plate received 2 mL of DAPI solution, which was then incubated at 37 °C for 15 min before washing with 1× PBS. The fluorescent nuclei were visualized under a microscope, and counting was performed using ImageJ software 13.0.5.

### 4.9. Generation of EVs-Treated bFGF

EVs were produced from WJ-MSCs using 2D and 3D culture methods with WT-bFGF (100-18B, Peprotech, Waltham, MA, USA) or TS-bFGF exosome-depleted 10% FBS medium. WJ-MSCs were grown in 150 mm dishes (20151, SPL) at a density of 1 × 10^6^ cells to generate 2D EVs. After reaching 80% confluence, the growth medium was replaced with α-MEM containing 10% EV-depleted FBS, and the cells were further cultured for 48 h. For advanced 3D culture, the WJ-MSCs were precoated with a rinsing solution (07010, STEMCELL Technologies, Vancouver, BC, Canada) on AggreWell400^TM^ plates (34425, STEMCELL Technologies). MSC spheroids were formed by incubating the plate overnight at 37 °C with 5% CO_2_. The generated MSC spheroids were transferred to 100 mm Petri dishes (10090, SPL) and placed on an orbital shaker at 60 rpm in α-MEM medium supplemented with 10% EV-depleted FBS. This rotation effectively maintained the spheroids. To produce bFGF-EVs and TS-3D EVs, 10 ng/mL exogenous bFGF were added to the medium. The supernatants were collected from the generated spheroids to isolate the EVs.

### 4.10. EVs Produced with WT-bFGF or TS-bFGF

Centrifugation was performed to isolate the EVs. The culture supernatant was centrifuged at 4 °C, 300× *g* for 10 min, then at 2000× *g* for 10 min, and lastly for 10,000× *g* to remove cell debris (Avanti J-E Centrifuge, Beckman Coulter). Lastly, EV precipitates were separated using ultracentrifugation at 178,000× *g* for 2 h at 4 °C (Optima L-90K, Beckman Coulter, Indianapolis, IN, USA). The EVs were measured at a concentration of 20–100 particles/frame after dilution in PBS. The NTA settings were as follows: focus, auto; shutter speed, 100; frames per second, 30; scattering intensity, detected automatically; and temperature, 25 °C. NTA was then performed using a 488 nm laser (ZetaView; TWIN PMX-220, Particle Metrix, Inning am Ammersee, Germany) after resuspension in 0.2 μm filtered phosphate-buffered saline (PBS, 10010023, Gibco).

### 4.11. Western Blotting

The protein levels were measured according to the manufacturer’s instructions for EV protein extraction. The EV samples were dissolved in RIPA buffer (CBR002, LPS solution) containing a protease inhibitor cocktail. Subsequently, the cell lysate was centrifuged at 13,000 rpm and 4 °C, and the resulting supernatant was transferred to a new tube. The EV proteins were quantified using the BCA protein assay kit (23225; Thermo Fisher Scientific, Waltham, MA, USA). The quantified EVs were transferred onto nitrocellulose membranes after electrophoresis on a 4–12% Bis-Tris Flus Gel (NW04125BOX, Invitrogen). The protein samples were blocked for 30 min and incubated with primary antibodies (1:1000) at 4 °C for one day. After washing thrice with 1× Tris-buffered saline with 0.1% Tween^®^ 20 detergent (TBST), the membranes were incubated with secondary antibodies for 2 h, followed by three additional washes with 1× TBST. Protein measurements were conducted using Invitrogen™ iBright™ Imagers (CL-1000) after processing the ECL substrate (170–5060, Bio-Rad Laboratories). The primary and secondary antibodies used were GAPDH (MA5-15738, Invitrogen), CD9 (ab263023, Abcam, Cambridge, UK), CD63 (ab109201, Abcam), CD81 (sc-7637, Santa Cruz Biotechnology, Dallas, TX, USA), GM130 (12480, Cell Signaling Technology (CST, Danvers, MA, USA), and calnexin (2679, CST). Horseradish peroxidase (HRP)-conjugated anti-rabbit IgG (7074, CST) and HRP-conjugated anti-mouse IgG (7076, CST) were the secondary antibodies.

### 4.12. In Vitro Migration Assay

We used the following method to evaluate exosome migration. HDF cells were seeded at 3 × 10^5^ cells/well in a 6-well plate and cultured until the cells reached approximately 95–100% confluency. Then, 10 μg/mL of mitomycin C (MMC, M4287, Sigma) diluted in DMEM-High was added to the well. After 2 h of MMC treatment, the cells were scraped vertically with a pipette tip and cultured with WT-bFGF or TS-bFGF EVs in the same medium. The cells were observed for 0, 12, 18, and 24 h. The migration area was then analyzed using ImageJ software 13.0.5 (National Institutes of Health, Bethesda, MD, USA).

### 4.13. NO Assay

The RAW 264.7 cells were seeded in a 24-well plate (30024, SPL) at 1.5 × 10^5^ cells/well and then incubated at 37 °C and 5% CO_2_ overnight. The cells were co-treated with DMEM-High containing LPS (L4391, Sigma), WT-bFGF, TS-bFGF EV, and 10% EV-depleted FBS. LPS (10 ng/mL), 10 μM of DEX, and 1 × 10^9^ particles/mL of EV were used during treatment. After 24 h of treatment, the supernatant of each sample was collected and centrifuged at 200× *g* for 3 min, and the supernatant was transferred to a new tube and stored at −80 °C. For NO measurement, solution 1, in which 0.1% N-(1-naphthyl) ethylenediamine dihydrochloride (33461, Sigma) was diluted in distilled water, and solution 2, in which 1% sulfanilamide (S9251, Sigma) was dissolved in 5% phosphoric acid, were used in a 1:1 ratio. After that, 100 μL of culture supernatant and 100 μL of reagent mixture were added to a 96-well plate and incubated at 25 °C for 10 min. The NO concentration was measured at 540 nm using a Bio-RADx-MarkTM spectrophotometer.

### 4.14. Statistical Analyses

GraphPad Prism software (version 9) was used to conduct all statistical analyses. All experiments were independently repeated thrice. Additionally, a one-way or two-way analysis of variance was conducted to determine the statistical significance in all figures. The p-values are indicated in each figure: *, **, ***, and **** represent *p* < 0.05, *p* < 0.01, *p* < 0.001, and *p* < 0.0001, respectively, to indicate the level of statistical significance. Furthermore, #, ##, ###, and #### represent statistical significance indicating *p* < 0.05, *p* < 0.01, *p* < 0.001, and *p* < 0.0001, respectively, compared to the WT-bFGF group.

## 5. Patent

We filed patents for this study (Korean patent application numbers 10-2023-0069536, 10-2023-0071436, and 10-2023-0071437).

## Figures and Tables

**Table 1:** Primers used for qRT-PCR.

Gene	Species	Accession No.	Primer Sequence(5′ to 3′)	Product Size (bp)
*GAPDH*	Human	NM_001357943.2	F: GTCTCCTCTGACTTCAACAGCG; R: ACCACCCTGTTGCTGTAGCCAA	131
*OCT4*	Human	NM_203289.6	F: CCTGAAGCAGAAGAGGATCACC; R: AAAGCGGCAGATGGTCGTTTGG	106
*SOX2*	Human	NM_003106.4	F: GCTACAGCATGATGCAGGACCA; R: TCTGCGAGCTGGTCATGGAGTT	135
*KLF4*	Human	NM_001314052.2	F: CATCTCAAGGCACACCTGCGAA; R: TCGGTCGCATTTTTGGCACTGG	156
*IL-1* *β*	Mouse	NM_008361.4	F: TGGACCTTCCAGGATGAGGACA; R: GTTCATCTCGGAGCCTGTAGTG	148
*TNF-* *α*	Mouse	NM_013693.3	F: GGTGCCTATGTCTCAGCCTCTT; R: GCCATAGAACTGATGAGAGGGAG	139

## Data Availability

Data are contained within the article.

## Supplementary Materials

The following supporting information can be downloaded from: https://www.mdpi.com/article/10.3390/ijms242216460/s1.
