# Supplementary material for: Thermostable Basic Fibroblast Growth Factor Enhances the Production and Activity of Human Wharton’s Jelly Mesenchymal Stem Cell-Derived Extracellular Vesicles
Source: Int J Mol Sci. 2023 Nov 17;24(22):16460. doi: 10.3390/ijms242216460 (PMC10671285; doi:10.3390/ijms242216460)
Supplement: Supplementary file 1 [file ijms-24-16460-s001.zip › ijms-2692416-supplementary.pdf]

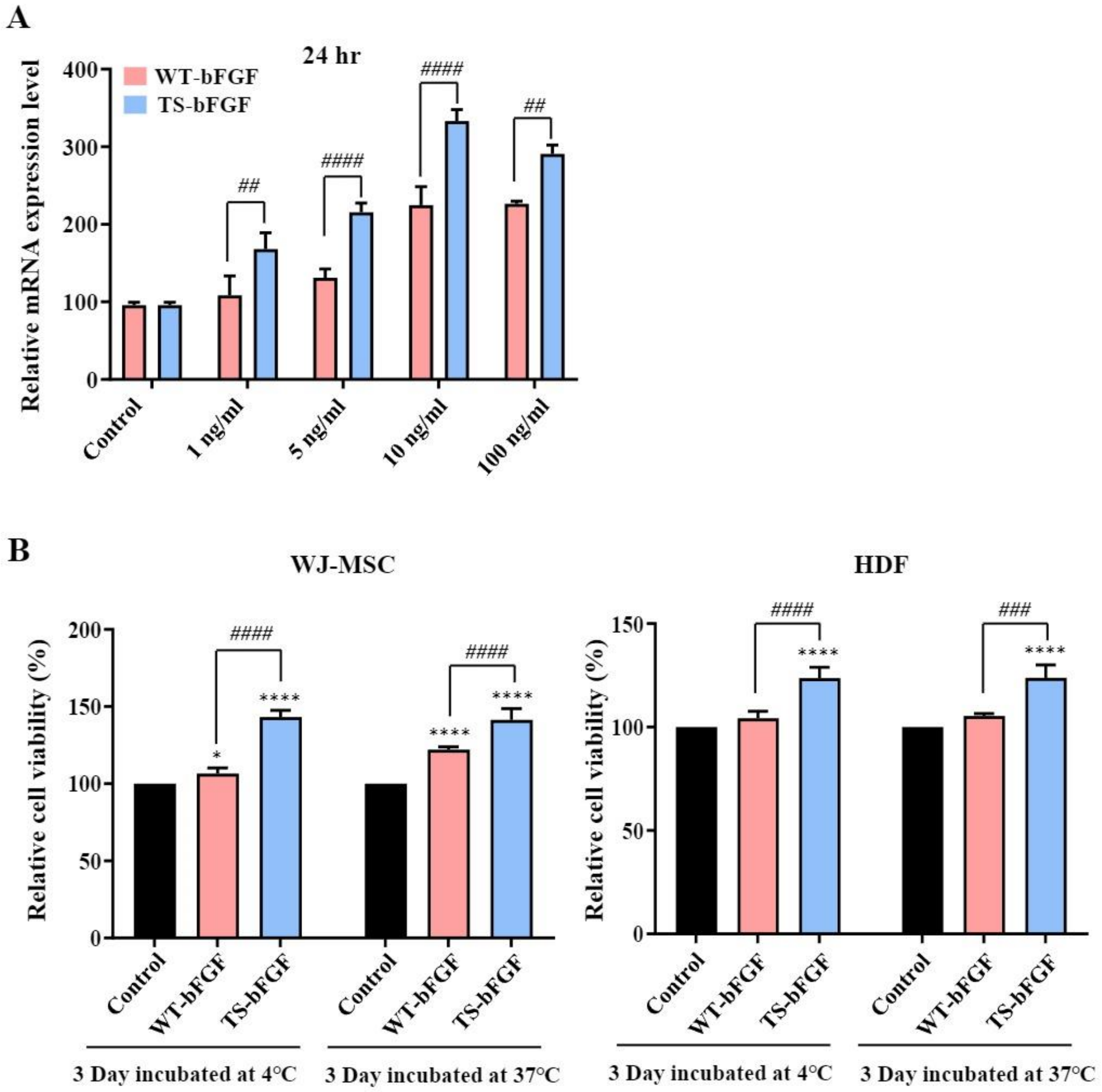

Figure S1. Temperature and Dose-dependent cell proliferation of WJ-MSCs treated with WT-bFGF or TS-bFGF

(A) In the comparison between groups treated with WT-bFGF and TS-bFGF at concentrations of 1, 5, 10, and 100 ng/mL after 24 hours, an increase in cell proliferation rate was observed as the concentration increased. (B) The cell proliferation rate was compared between WJ-MSCs and HDFs after treatment with bFGF at both 4 °C and 37 °C. The results showed that the TS-bFGF-treated group exhibited a higher cell proliferation rate than the WT-bFGF-treated group at both 4 °C and 37 °C (p-value * < 0.05, ** < 0.01, **** < 0.0001 vs. Control; ## < 0.01, ### < 0.001, #### < 0.0001 vs. WT-bFGF).

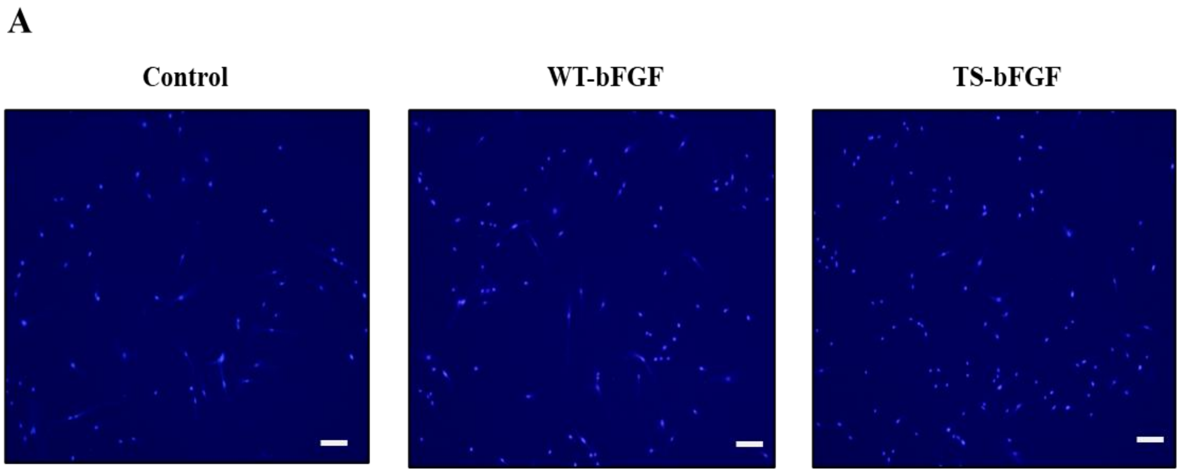

Figure S2. DAPI-stained Images of WJ-MSCs treated with WT-bFGF or TS-bFGF

(A) Photographic images after treatment with WT-bFGF and TS-bFGF (Scale bar represents 200 μm).

Supplementary Table S1. Exosome production of WJ-MSCs under various culture conditions

	2D WJ-MSCs	3D WJ-MSCs	WT-3D WJ-MSCs	TS-3D WJ-MSCs
Culture volume (mL)	60	100	100	100
Total number of cells	1.54×10^{7}	1.74×10^{7}	2.44×10^{7}	2.32×10^{7}
Total number of EVs (purified)	1.44×10^{10}	4.26×10^{10}	1.14×10^{11}	1.39×10^{11}
Total number of EVs (conditioned cell culture media)	3.1×10^{11} (5.2×10^{9}/mL culture)	8.3×10^{11} (8.3×10^{9}/mL culture)	2.3×10^{12} (2.3×10^{10}/mL culture)	3.0×10^{12} (3.0×10^{10}/mL culture)
